# Correction of Liver Steatosis by a Hydrophobic Iminosugar Modulating Glycosphingolipids Metabolism

**DOI:** 10.1371/journal.pone.0038520

**Published:** 2012-10-08

**Authors:** Elisa Lombardo, Cindy P. A. A. van Roomen, Gijs H. van Puijvelde, Roelof Ottenhoff, Marco van Eijk, Jan Aten, Johan Kuiper, Herman S. Overkleeft, Albert K. Groen, Arthur J. Verhoeven, Johannes M. F. G. Aerts, Florence Bietrix

**Affiliations:** 1 Departments of Medical Biochemistry, Academic Medical Center, University of Amsterdam, Amsterdam, The Netherlands; 2 Division of Biopharmaceutics, Leiden/Amsterdam Center for Drug Research (LACDR), Leiden University, Leiden, The Netherlands; 3 Department of Pathology, Academic Medical Center, University of Amsterdam, Amsterdam, The Netherlands; 4 Division of Biopharmaceutics, Leiden Institute of Chemistry, Leiden University, Leiden, The Netherlands; 5 Department of Pediatrics, University Medical Center Groningen, Groningen, The Netherlands; Copenhagen University Hospital Gentofte, Denmark

## Abstract

The iminosugar N-(5′-adamantane-1′-yl-methoxy)-pentyl-1-deoxynoijirimycin (AMP-DNM), an inhibitor of glycosphingolipid (GSL) biosynthesis is known to ameliorate diabetes, insulin sensitivity and to prevent liver steatosis in ob/ob mice. Thus far the effect of GSL synthesis inhibition on pre-existing NASH has not yet been assessed. To investigate it, LDLR(−/−) mice were kept on a western-type diet for 12 weeks to induce NASH. Next, the diet was continued for 6 weeks in presence or not of AMP-DNM in the diet. AMP-DNM treated mice showed less liver steatosis, inflammation and fibrosis. Induction of fatty acid beta-oxydation was observed, as well as a reduction of plasma lipids. Our study demonstrates that AMP-DNM treatment is able to significantly correct pre-existing NASH, suggesting that inhibiting GSL synthesis may represent a novel strategy for the treatment of this pathology.

## Introduction

The metabolic syndrome represents a combination of health risk factors including abdominal obesity, insulin resistance, dyslipidemia and hypertension. Non Alcoholic Fatty Liver Disease (NAFLD) is the hepatic manifestation of the metabolic syndrome. NALFD includes a large variety of liver derangements ranging from simple fat accumulation in the parenchymal cells (steatosis) to non-alcoholic steatohepatitis (NASH) including inflammation and varying degrees of fibrosis. NAFLD is estimated to affect at least 20% of the general adult population and over 50% of the obese population [1], [2]. In about 30% of NAFLD patients, the disease can progress into steatohepatitis and cirrhosis [3]. It is expected that as the prevalence of obesity and metabolic syndrome rises, NAFLD-associated diseases will be an increasing healthcare concern and therapeutic measures are thus needed to address this major health problem [4], [5]. Fat accumulation in hepatocytes is the result of an imbalance between triglyceride synthesis and degradation. An increased flux and/or endogenous synthesis of free fatty acids (FFA) may lead to accumulation of triglycerides within the liver when mitochondrial β-oxidation and VLDL production and secretion are not sufficient to handle the FFA load. The molecular mechanisms behind this fat accumulation in hepatocytes leading to NASH still remain unclear. Hepatic inflammatory cell recruitment and inflammatory cytokines appear to play a key role in this process and dietary cholesterol has been proposed to be an important contributor for the development of the pathology in hyperlipidemic mouse models [6], [7].

We and others have previously shown that two distinct classes of inhibitors of glucosylceramide (GlcCer) synthase, the rate limiting enzyme involved in glycosphingolipid (GSL) biosynthesis, improved glycemic control, decreased insulin resistance and reduced fatty liver development in animal models of obesity i.e. diet-induced obesity (DIO) mice and ob/ob mice [8]–[13]. A particular role for the ganglioside GM3 in insulin sensitivity has become apparent in recent times. Firstly, Yamashita et al. reported that mice deficient in GM3 synthase, and thus deficient in the ganglioside GM3 and higher gangliosides like GM2-glycol, are protected against insulin resistance and obesity [14]. Inokuchi and co-workers showed that the ganglioside GM3 interacts directly with a lysine residue in the insulin receptor [15]. The role of gangliosides in insulin sensitivity has recently been reviewed [16], [17]. The use of the iminosugar N-(5′-adamantane-1′-yl-methoxy)-pentyl-1-deoxynoijirimycin (AMP-DNM) and ceramide-mimic Genz-123346 [(1R,2R)-nonanoic acid[2-(2′,3′-dihydro-benzo [1], [4] dioxin-6′-yl)-2-hydroxy-1-pyrrolidin-1-ylmethyl-ethyl]-amide-L-tartaric acid salt], both inhibitors of GlcCer synthase, clearly improved liver steatosis [13]. However, the ability of AMP-DNM to correct liver steatosis and even NASH when it already has developed, has not yet been investigated.

In the present study, LDLR(−/−) and APOE*3 Leiden mice, two models sensitive to liver steatosis were allowed to develop NASH for 12 weeks on high fat-high cholesterol diet and were subsequently treated with AMP-DNM for 6 weeks. We observed that despite the maintenance of the animals on a high fat-high cholesterol diet, AMP-DNM treatment reduced plasma lipids and that the steatosis, the inflammatory and fibrotic status were profoundly improved.

## Results

### AMP-DNM treatment ameliorates hyperlipidemia and reverses hepatic steatosis in LDLR(−/−) mice

In the present study, 40 LDLR(−/−) mice were fed a western-type diet to induce NASH and were subsequently treated with two different doses of AMP-DNM to achieve the dosing level of 50 and 100 mg AMP-DNM. kg bw^−1^.day^−1^. AMP-DNM supplementation did not affect the behaviour of the animals. At the dose of 100 mg AMP-DNM, the bodyweight of the animals was reduced compared to the control group and food consumption slightly decreased after the switch of diet (table S1). This was not observed in the group treated with 50 mg AMP-DNM. AMP-DNM treatment induced a dose-dependent decrease of plasma GlcCer and ceramide (table 1). Whereas the amount of GlcCer was dose-dependently decreased in the livers of treated animals, ceramide amounts were not changed (table 1). AMP-DNM also dose-dependently decreased plasma triglycerides, FFA, and cholesterol (fig. 1A–C). We also determined the effect of AMP-DNM treatment on hepatic concentration of higher glycosphingolipids: lactosylceramide (Laccer); globotriaosylceramide (Gb3) and gangliosides (table 2). The neutral glycosphingolipids LacCer and Gb3 were reduced by AMP-DNM treatment. The most abundant ganglioside was GM2-glycol. It was significantly reduced by drug treatment. This was not seen for the less abundant GM3.

**Figure 1:**
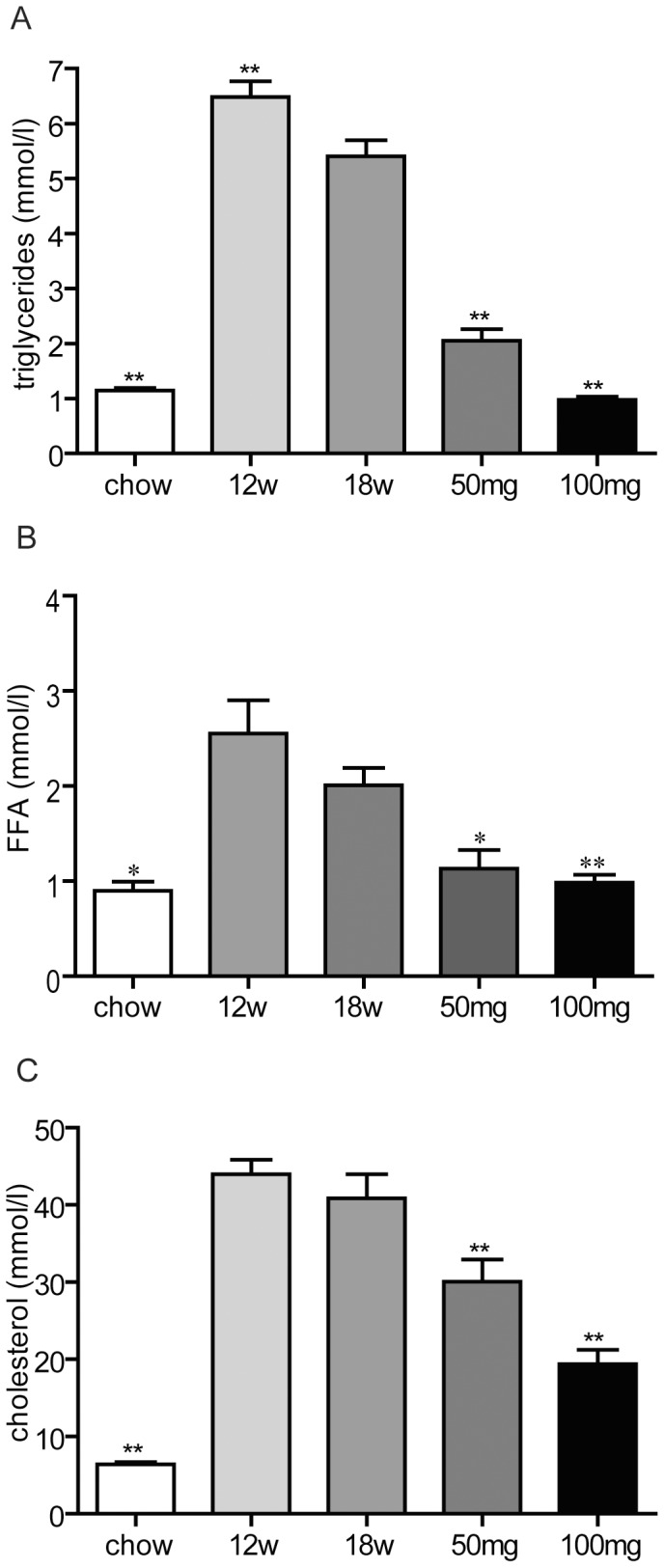
Effect of AMP-DNM treatment on plasma lipids in LDLR(−/−) mice. Mice were fed a western-type diet for 18 weeks, and received in the last 6 weeks either 0, 50 or 100 mg AMP-DNM. Plasma concentration of triglycerides (A), free fatty acids (FFA) (B) and cholesterol (C) after 18 weeks of chow diet (chow), 12 weeks of western-type diet (12w) or 18 weeks of western-type diet with or without AMP-DNM treatment at the indicated doses. *p<0.05; **p<0.01.

**Table 1:** Plasma and liver glucosylceramide (glccer) and ceramide (cer) concentrations in LDLR(−/−) mice fed a western-type diet for 18 weeks, receiving in the last 6 weeks either 0, 50 or 100 mg AMP-DNM.

	plasma (nmol.ml^−1^)		liver (nmol.g^−1^ liver)	
	glccer	cer	glccer	cer
**chow**	11.5±0.3**	6.7±0.4**	38.5±7.0	123.1±11.2
**12w**	81.4±4.6	45.2±5.2	46.1±3.8	131.9±5.8
**18w**	85.5±6.7	45.1±2.6	62.5±5.5	150.8±7.7
**50 mg**	32.0±3.2**	35.3±4.7	33.4±3.9***	150.1±11
**100 mg**	10.1±0.8**	17.7±0.7**	21.7±1.3***	161.7±8

Data are expressed as mean ± SEM of 5 (plasma) and 10 (liver) mice.

*** , p<0.001.

**Table 2:** Liver lactosylceramide (Laccer) and globotrihexosylceramide (Gb3) and gangliosides (GM2-gl and GM3) concentrations in LDLR(−/−) mice fed a western-type diet for 18 weeks, receiving in the last 6 weeks either 0, 50 or 100 mg AMP-DNM.

	liver (nmol.g^−1^ liver)			
	Laccer	Gb3	GM2-gl	GM3
**12w**	16.4±1.3	7.2±0.6	255.9±19.6	19.8±8.5
**18w**	22.8±1.2	6.3±0.8	308.7±22.5	23.2±3.4
**50 mg**	16.5±1.1**	2.8±0.3**	338.8±14.5	24.6±4.1
**100 mg**	14.6±1.5***	0.9.±0.1***	173.4±13.8***	16.7±2.6

Data are expressed as mean ± SEM of 5 mice.

** , p<0.01;

*** , p<0.001.

We then determined the effect of AMP-DNM on liver steatosis. First liver weight was significantly reduced in a dose-dependent fashion (25% reduction with 50 mg AMP-DNM and 32% reduction with 100 mg AMP-DNM, p<0.01). Analyses of morphology and fat content on liver sections stained with H&E (fig. 2A) and Oil-red-O (fig. 2B) showed that after 12 weeks on western-type diet feeding, livers of LDLR(−/−) contained micro and macro-lipid droplets, which were slightly increased in number and in size after 18 weeks of diet. By contrast, the animals treated with 50 mg AMP-DNM showed a dramatic reduction in the amount of hepatic lipid droplets, whereas at the dose of 100 mg AMP-DNM, virtually no lipid droplets were present. Biochemical analysis of lipids confirmed a dose-dependent reduction of hepatic triglycerides (fig. 2C). Free cholesterol content was significantly reduced at the dose of 100 mg AMP-DNM (fig. 2D).

**Figure 2:**
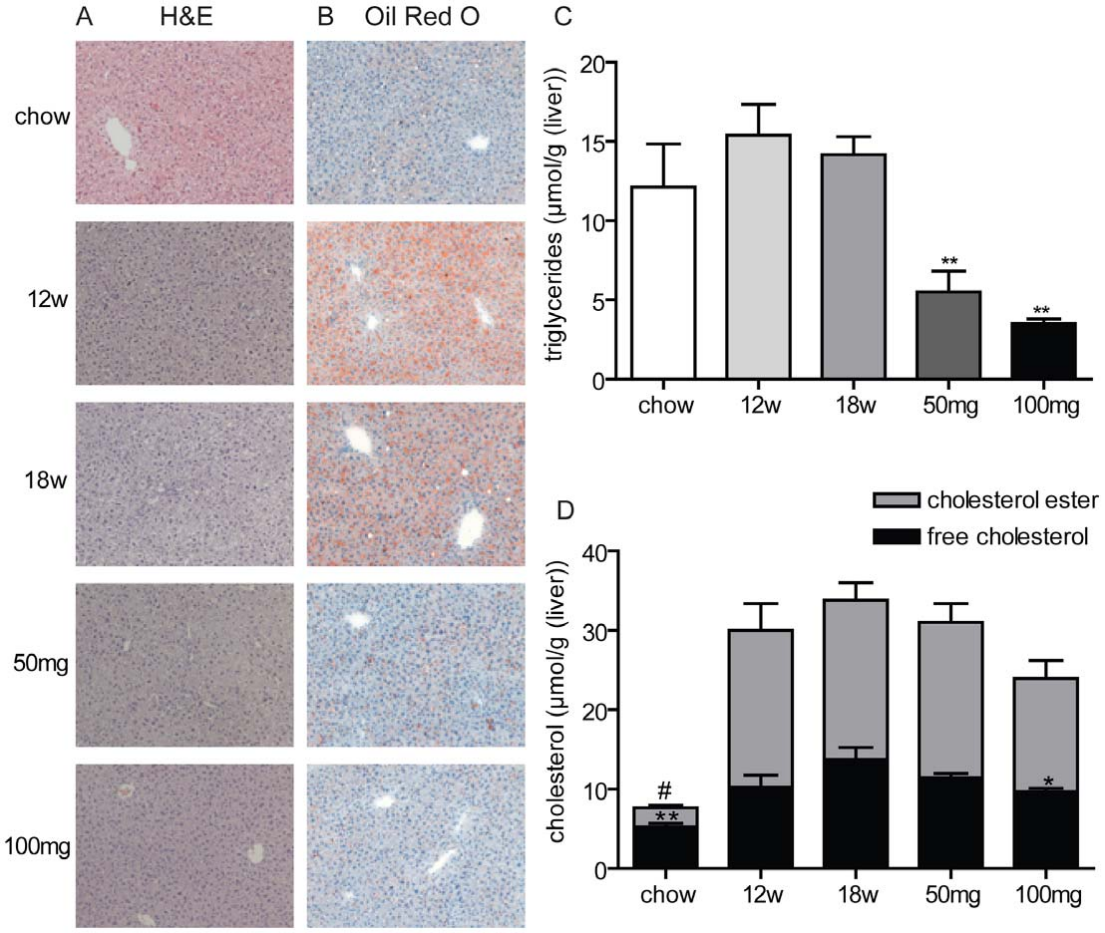
Effect of AMP-DNM treatment on liver steatosis in LDLR(−/−) mice. Mice were fed a western-type diet for 18 weeks, and received in the last 6 weeks either 0, 50 or 100 mg AMP-DNM. (A) Representative photomicrographs of hematoxylin-eosin staining and Oil red O staining (B) of livers section after 18 weeks of chow diet (chow), 12 weeks of western-type diet (12w) or 18 weeks of western-type diet with or without AMP-DNM treatment at the indicated doses (original magnification ×10). (C) Triglyceride content and (D) cholesterol content in liver of animals for the indicated groups. *p<0.05; **p<0.01. #p<0.01 for esterified cholesterol.

### AMP-DNM treatment modulates hepatic lipogenesis and glucose production in LDLR(−/−) mice

AMP-DNM ameliorates glucose homeostasis and insulin sensitivity in obese rodents [9], [11], [12], [18]. In LDLR(−/−) animals, we observed that blood glucose levels were significantly lower at the end of the treatment compared to control animals (fig. 3A). Insulin levels also tend to decrease with the treatment (fig. 3B). The homeostasis model assessment (HOMA) index was significantly reduced in treated animals (fig. 3C). As a consequence of the improved glucose homeostasis, the percentage of glycated haemoglobin was notably reduced as well (fig. 3D).

**Figure 3:**
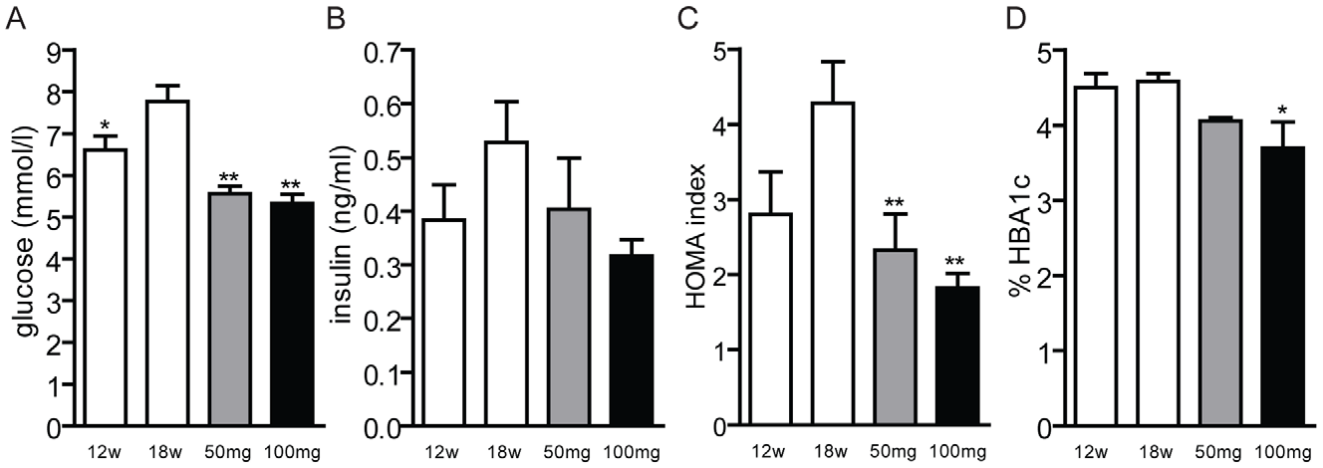
Effect of AMP-DNM treatment on insulin sensitivity in LDLR(−/−) mice. Mice were fed a western-type diet for 18 weeks, and received in the last 6 weeks either 0, 50 or 100 mg AMP-DNM. (A) Glucose concentration and (B) insulin concentration in plasma of fasted animals. (C) Homeostasis model assessment (HOMA) index and (D) percentage of glycated heamoglobin 1c (HbA1_c_). *p<0.05; **p<0.01.

Sterol regulatory element binding protein 1c (SREBP1c) is a transcription factor activated by insulin and responsible for the transcription of numerous genes involved in fatty acid synthesis. Despite insulin resistance for the glucose homeostasis pathway, it has been reported that hyperinsulinemia triggers the activation of SREBP-1c and consequently fatty acid synthesis [19]. As anticipated, we observed that the western-type diet induced an up-regulation of SREBP1 (fig. 4) and some of its target genes, i.e. fatty acid synthase (FAS) and stearoyl-CoA desaturase (SCD1). AMP-DNM treatment normalized expression of these genes. Moreover, we observed that the western-type diet induced expression of PDK4 and glucose-6-phosphatase. Animals treated with AMP-DNM showed corrected expression of both genes. Of interest, CPT1a and PCG1a were increased by AMP-DNM treatment and levels of LCA mRNA tended to be also higher with drug treatment at the highest dose (fig. 4). These findings suggest that fatty acid beta-oxidation is increased by AMP-DNM treatment. Taken together, our data strongly suggest that the treatment with AMP-DNM increases insulin sensitivity in the livers of treated animals, reduces lipogenesis and promotes fatty acid beta-oxidation.

**Figure 4:**
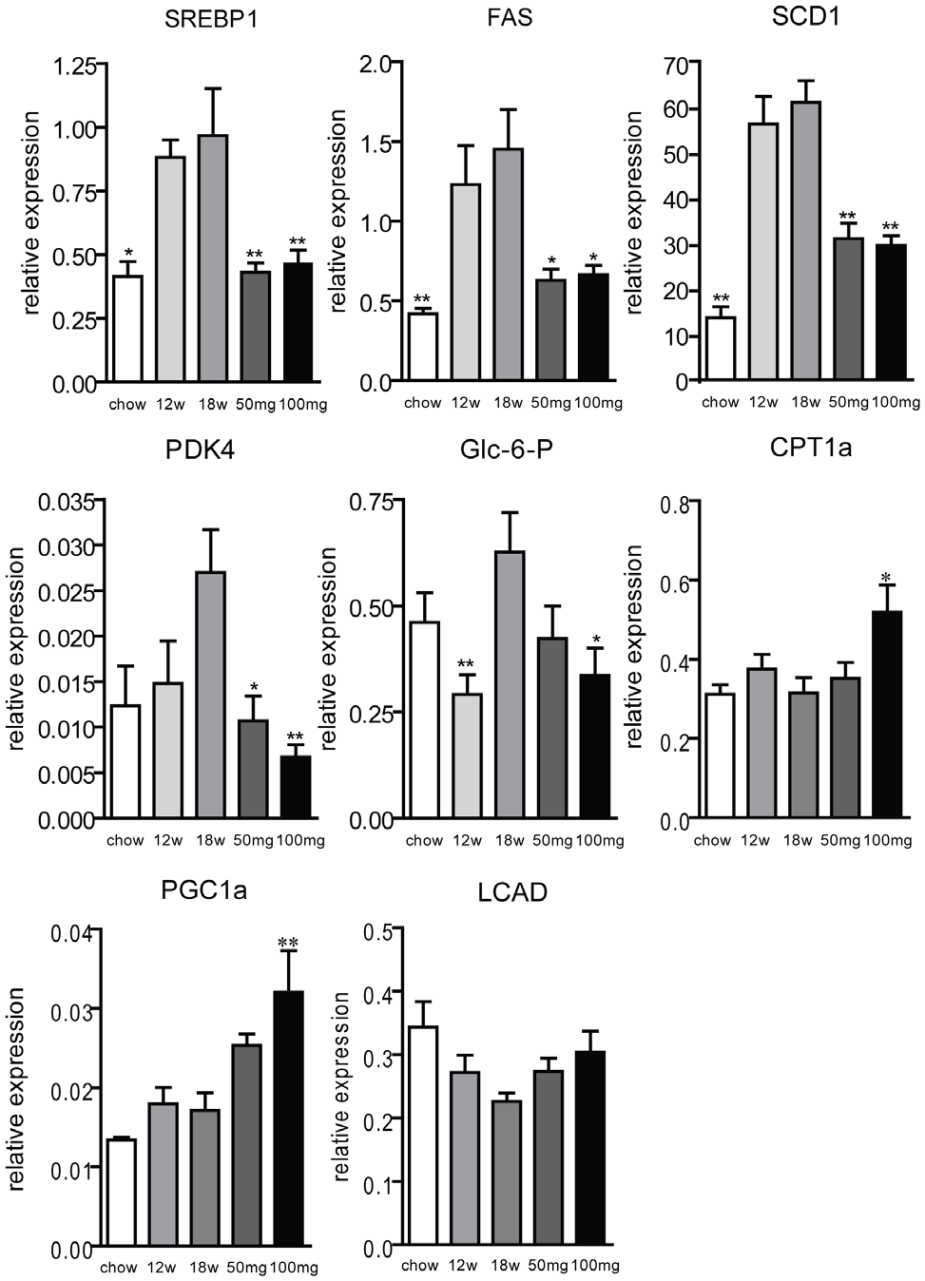
Effect of AMP-DNM treatment on genes involved in lipid metabolism in livers of LDLR(−/−) mice. Mice were fed a western-type diet for 18 weeks, and received in the last 6 weeks either 0, 50 or 100 mg AMP-DNM. Expression levels normalized to Acidic ribosomal phosphoprotein (36B4). *p<0.05; **p<0.01.

### AMP-DNM treatment reduces hepatic inflammation and fibrosis

Next, we examined the effect of AMP-DNM treatment on hepatic inflammation (fig. 5). Detailed immunohistochemical analysis on liver sections stained with the macrophage marker CD68 (Fig. 5A) revealed that after 12 and 18 weeks of western-type diet, the Kuppfer cells (the resident macrophages of liver) became foamy. At the dose of 50 mg AMP-DNM, Kuppfer cells showed an intermediate phenotype with a reduction of their size. At the dose of 100 mg AMP-DNM, Kuppfer cells looked even closer to normal, almost equal in size to those in animals on chow diet.

**Figure 5:**
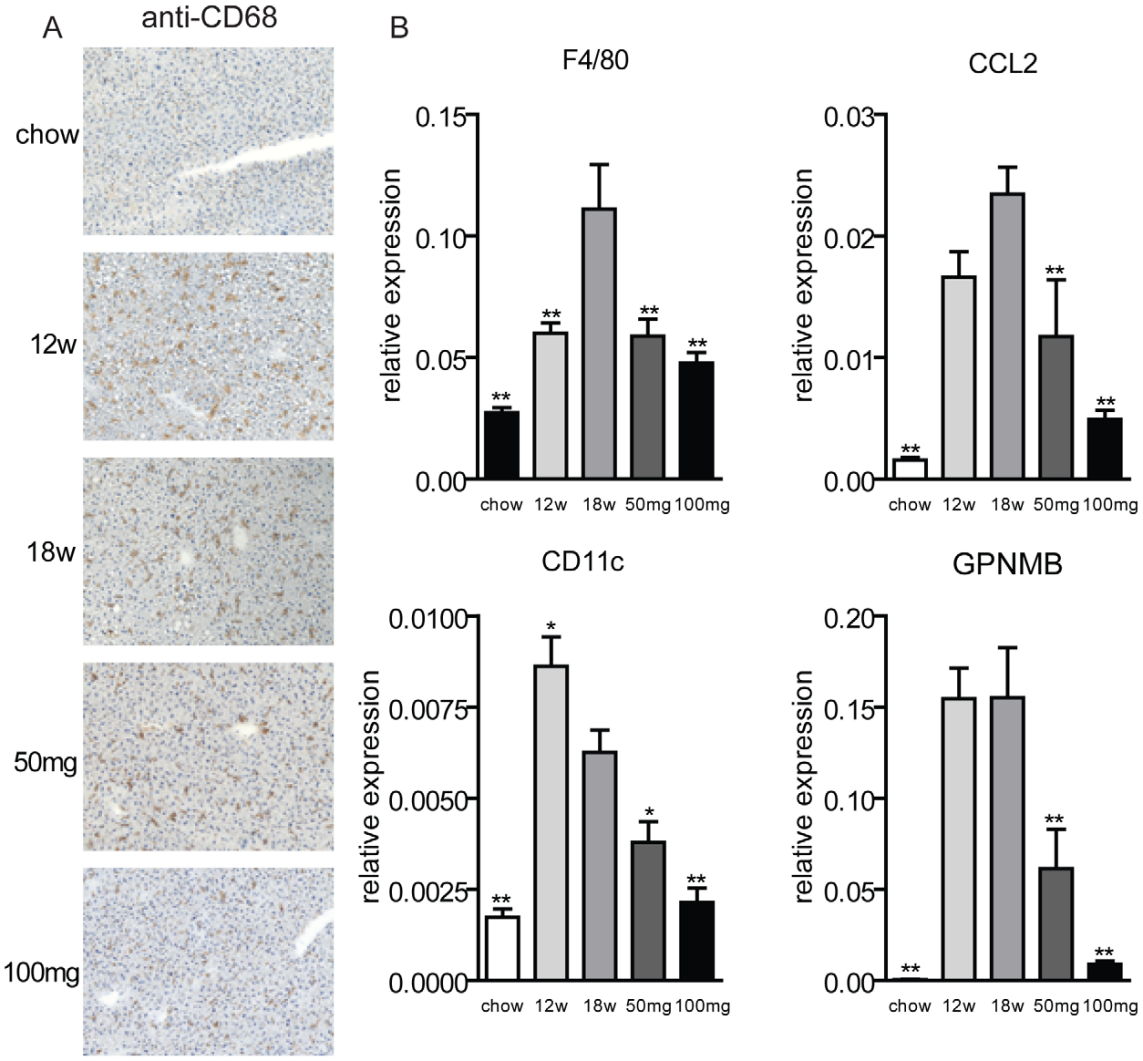
Effect of AMP-DNM treatment on hepatic inflammation in LDLR(−/−) mice. Mice were fed a western-type diet for 18 weeks, and received in the last 6 weeks either 0, 50 or 100 mg AMP-DNM. (A) Representative photomicrographs of immunohistochemical staining of CD68 of the indicated mice (original magnification ×10). (B) Gene expression of inflammation markers. *p<0.05; **p<0.01.

Gene expression profiles of inflammatory markers were also monitored (fig. 5B). After 18 weeks of western-type diet the expression of the macrophage marker F4/80 was increased 4-fold, the chemokine (C-C motif) ligand 2 (CCL2)/monocyte chemoattractant protein 1 (MCP-1) 15-fold, and CD11c 3-fold.

We also analysed expression levels of Glycoprotein Nonmetastatic Melanoma Protein B (GPNMB) also called osteoactivin, a new marker of monocyte to macrophage differentiation [20]. On normal chow diet, GPNMB was poorly expressed but feeding with the western-type diet resulted in a strong up-regulation of its expression (i.e. 300-fold increase after 18 weeks of western-type diet). AMP-DNM treatment resulted in a dose-dependent reduction of expression of each of these markers. At the dose of 100 mg AMP-DNM, the reduction was even larger. It suggests that the highest dose of AMP-DNM completely corrected the effect of the western-type diet on liver inflammation.

An important feature in NASH is the progression towards liver fibrosis. To monitor this aspect, we stained sections with Sirius-red. In all groups of animals, positive collagen staining was observed and was localized near vessels of periportal and centrolobular regions. After 12 and 18 weeks of western-type diet, collagen staining in these two areas was higher than in animals on chow diet. Collagen content was slightly reduced as a consequence of AMP-DNM treatment (fig. 6A). Immunohistochemistry staining of activated stellate cells with anti-SMA antibody and gene expression analysis of SMA confirmed this finding (fig. 6B, C). Gene expression profiles of the collagen 1 alpha (col 1α) and transforming growth factor beta (TGFβ) were also monitored (fig. 6C). Both these markers were considerably increased with the western-type diet, whereas the AMP-DNM treatment normalized their expressions levels.

**Figure 6:**
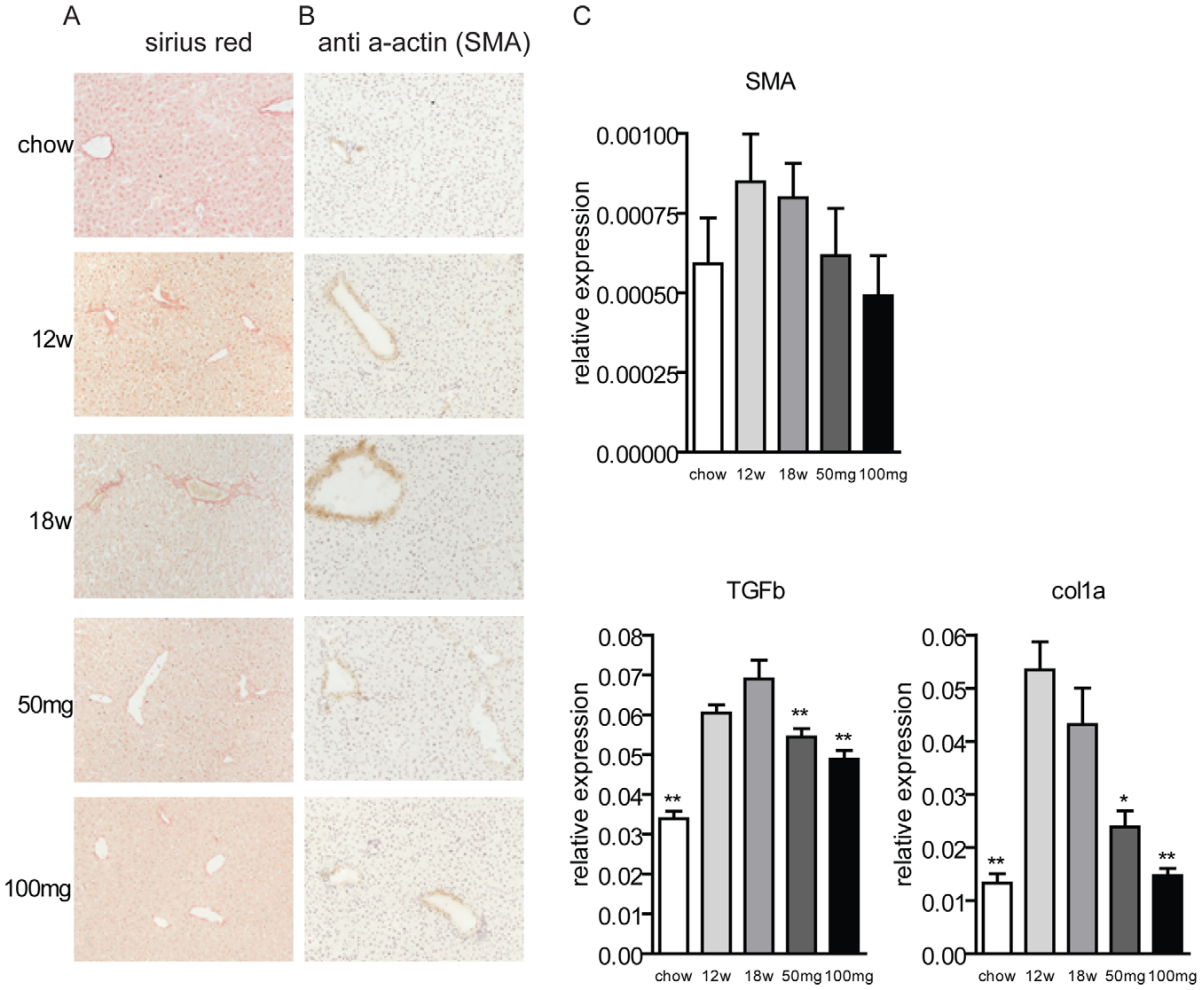
Effect of AMP-DNM treatment on liver fibrosis in LDLR(−/−) mice. Mice were fed a western-type diet for 18 weeks, and received in the last 6 weeks either 0, 50 or 100 mg AMP-DNM. (A) Representative photomicrographs of Sirius-red staining of liver sections of the indicated mice (original magnification ×10). (B) Representative photomicrographs of immunohistochemical staining of activated stellate cells (original magnification ×10). (C) Gene expression of fibrosis marker. *p<0.05; **p<0.01.

### AMP-DNM also corrects hepatic steatosis in APOE*3 Leiden

We examined also the impact of AMP-DNM treatment on fatty liver of APOE*3 Leiden mice that were exposed for 12 weeks to a 1% cholesterol, 15% fat diet. The switch of diet induced a slight reduction of the animal bodyweights treated with either 50 or 100 mg AMP-DNM. No major changes were observed concerning the food consumption (table S2). The liver of these animals after 12 weeks of diet showed steatosis, but no clear fibrosis as compared to the LDLR(−/−) mice (fig. 7A). Animals were subsequently switched for 6 weeks to 0.25% cholesterol, 15% fat diet with or without AMP-DNM. The drug treatment lowered as expected the levels of glucosylceramide in plasma and liver of treated animals (tables S3 and S4) and it dramatically corrected liver steatosis that was still prominent in the control animals (fig. 7A). AMP-DNM also resulted in corrections in liver triglyceride and cholesterol content (fig. 7B).

**Figure 7:**
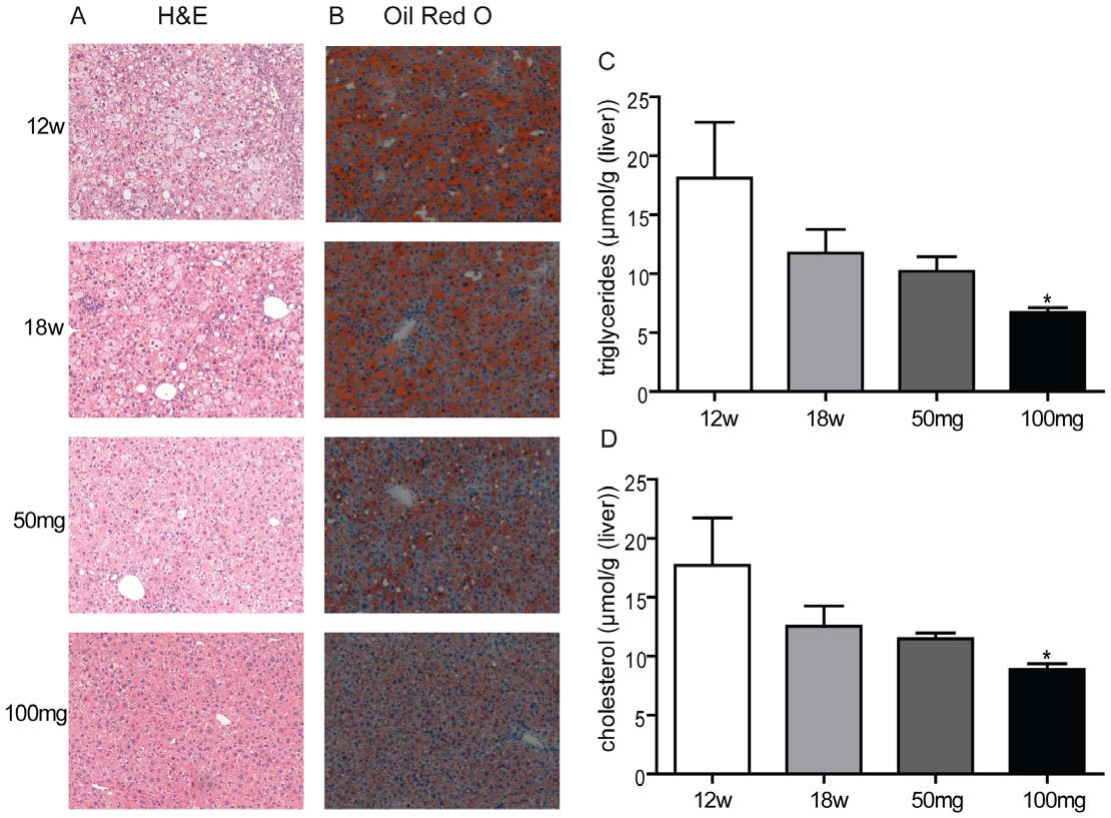
Effect of AMP-DNM treatment on liver steatosis in APOE*3 Leiden mice. Mice were fed a high cholesterol-high fat diet (1% cholesterol, 15% fat) for 12 weeks and were fed for 6 weeks more a western-type diet (0.25% cholesterol, 15% fat) supplemented with either 0, 50 or 100 mg AMP-DNM. Representative photomicrographs of hematoxylin-eosin staining (A) and Oil red O staining (B) of livers section after 12 weeks of high cholesterol-high fat diet (12w) or after 12 weeks of high fat-high cholesterol diet followed by 6 weeks of western-type diet (18w) with or without AMP-DNM treatment at the indicated doses (original magnification ×10). (C) Triglycerides content and (D) cholesterol content in liver of animals for the indicated groups. *p<0.05; **p<0.01.

## Discussion

We have previously demonstrated in leptin-deficient ob/ob mice that AMP-DNM treatment reduced liver steatosis associated with a restoration of liver insulin signaling [9]. Also hepatic fatty acid synthesis and glycogen storage were normalized. Two other inhibitors of GlcCer synthase, Genz-123346 and Genz-112638, also corrected liver steatosis in diet-induced obese (DIO) mice [10], [13].

A limitation of the leptin-deficient ob/ob and DIO models is that the mice do not develop steatohepatitis or liver fibrosis as is observed in humans [21]. LDLR(−/−) mice fed a western-type diet consisting of a moderate amount of fat (15%) and cholesterol (0.25%) offer a better model to investigate NASH. The animals, showing a human “like” lipoprotein profile, rapidly develop hepatic steatosis and inflammation [22]. We have previously observed in this model that treatment with AMP-DNM from the start of the diet reduces liver triglyceride content [23]. To study the ability of AMP-DNM to also correct hepatic manifestations of the metabolic syndrome, LDLR(−/−) mice were kept on a western-type diet for 12 weeks to induce NASH. Next, the diet was continued for 6 weeks in the presence or absence of AMP-DNM in the diet. Our study demonstrates that AMP-DNM treatment is not only able to prevent liver steatosis, but is also able to significantly correct pre-existing NASH. AMP-DNM treated mice showed less liver steatosis and inflammation with a liver phenotype closely resembling that of matched LDLR(−/−) mice fed a standard chow diet. In APOE*3 Leiden mice, another hyperlipidemic model sensitive to hepatic steatosis, AMP-DNM treatment corrected liver accumulation of fat and reduced inflammation in a similar fashion to what was observed with LDLR(−/−) mice. In this experiment set-up, animals were first fed with a high-fat high cholesterol diet (1% cholesterol, 15% fat) for 12 weeks and fed for 6 following weeks a western-type diet (0.25% cholesterol, 15% fat) supplemented with either 0, 50 or 100 mg AMP-DNM. It has to be noted that the decrease in cholesterol in the diet tends by itself to decrease liver lipids in the control animals (table S3) whereas we observed a poor effect of the reduction of lipids in the diet on plasma cholesterol and triglycerides. The AMP-DNM treatment induced a significant and prominent decrease of these parameters in both compartment.

The underlying mechanism by which AMP-DNM treatment is able to correct NASH manifestation is intriguing. In the LDLR(−/−) mice the most prominent ganglioside in liver is GM2-glycol, rather than GM3. AMP-DNM treatment led to a major reduction in GM2-glycol, accompany the correction of liver steatosis. It may be speculated that GM2-glycol, rather than GM3, causes insulin resistance in the LDLR(−/−) mice liver. Hyperinsulinemia has been proposed to promote liver steatosis [19] and hyperinsulinemic patients with NASH treated with insulin-sensitizing agents show improvements of liver steatosis [24]. It is well known that inhibitors of GlcCer synthase like AMP-DNM and Genz-123346 are also able to improve insulin sensitivity in various rodent models by virtue of lowering glycosphingolipids [16], [17]. LDLR(−/−) mice treated with AMP-DNM in this study showed an improvement of the HOMA index and a concomitant decrease of hepatic lipogenesis and increased beta-oxidation. Recent experiments with ob/ob mice also revealed that AMP-DNM causes major changes in hepatic lipid metabolism. The rates of fatty acid beta-oxidation and glucose oxidation were determined using metabolic cages, revealing that shortly after exposure to AMP-DNM, fat oxidation was nearly doubled (0.07 to 0.16 kcal.h^−1^ in the nocturnal period and 0.10 to 0.19 kcal.h^−1^ in the diurnal period). On the opposite, carbohydrate oxidation was markedly reduced by AMP-DNM treatment (0.43 to 0.28 kcal.h^−1^ in the nocturnal period and 0.34 to 0.20 kcal.h^−1^ in the diurnal period [25]. Other researchers have also reported beneficial effect of iminosugars on hepatic fatty acid oxidation. Similar to our findings with AMP-DNM, Tsuduki et al reported that intake of 1-deoxynojirimycin promotes hepatic beta-oxidation and suppresses lipid accumulation in rat liver [26]. Kobayashi and co-worker reported similar effects induced by mulberry extracts known to be rich in simple iminosugars [27]. It is likely that these metabolic changes, following improved insulin sensitivity, contribute to the lowering of triglycerides in hepatocytes and the correction of steatosis observed in LDLR(−/−) and APOE*3 Leiden mice (figure S1 and S2).

Of note, lowering of glycosphingolipids by specific genetic knock-down of GlcCer synthase in hepatocytes does not reduce liver triglycerides in mice exposed to a high fat diet [28]. It seems unlikely that the insulin-sensitizing effect of AMP-DNM alone explains the observed effects on hepatic inflammation and fibrosis. Presumably the earlier described anti-inflammatory actions of AMP-DNM play a key role as well [12], [29].

In the past years, several studies showed that ER stress response plays an important role in lipid metabolism and is linked to fatty liver disease [30]–[32]. The role of glycosphingolipids in ER stress response has not been extensively studied yet. A recent study on pancreatic MIN6 β-cell has revealed that ER stress is associated with increased GlcCer and that it is ameliorated by transfection of cells with glucosylceramide synthase [33]. Ceramide concentration is not changed. In our previous study on AMP-DNM treatment of ob/ob mice, microarray data for liver did not reveal any significant changes in expression of proteins involved in pathways associated with ER stress [9]. It nevertheless remains of interest to study in more detail the possible role of (glyco)sphingolipids in ER stress responses and related effects on lipid homeostasis.

Liver natural killer T (NKT) cells are thought to be able to ameliorate steatosis and NKT cells numbers are reduced in steatotic livers of human subjects and ob/ob mice [34], [35]. We anticipated that AMP-DNM treatment might modulate the hepatic NKT cell population. Non-parenchymal cells were therefore isolated from livers of treated animals and screened for NKT markers by flow cytometry (fig. S3A). The percentage of hepatic NKT cells upon AMP-DNM treatment did not change, suggesting that this cell population was not causing the observed correction of NASH. CD4+ - and CD8+ T-cell populations were also unaffected by AMP-DNM (fig. S3B).

AMP-DNM exerts a positive effect on cholesterol homeostasis by promoting biliary sterol secretion and fecal excretion [23]. Increased hepatic free cholesterol in obese, diabetic mice is a known critical factor in the development of nonalcoholic steatohepatitis [36], [37]. Of interest, AMP-DNM treatment at the highest dose of 100 mg. kg bw^−1^. day^−1^ led to a significant decrease in free cholesterol content of liver of the LDLR(−/−) mice. We also observed a strong dose-dependent reduction of plasma cholesterol in LDLR(−/−) animals treated with AMP-DNM. All this was accompanied by reduction of F4/80 positive cells in the liver (fig. S3C). AMP-DNM treatment clearly decreased (macrophage) inflammation in the liver. The positive effects on cholesterol homeostasis exerted by AMP-DNM may contribute to the reversal in hepatosteatosis. High cholesterol in the western-type diet is detrimental since it drives inflammation due to uptake of modified cholesterol-rich lipoproteins by Kuppfer cells [38]. We therefore would like to propose that AMP-DNM, by improving cholesterol homeostasis, causes a beneficial reduction of Kuppfer cell activation.

In conclusion, our study shows clearly that drug treatment is sufficient to swiftly correct the liver manifestations of NASH in two different animal models with improved insulin sensitivity, promotion of fatty acid beta-oxidation and reduction of inflammation.

## Materials and Methods

### Materials

AMP-DNM was synthesized as previously described [39], [40]. All solvents and reagents used were of analytical grade.

### Mice and diets

Experiments were performed with the approval of the local Ethical Committee for Animal Experiments.

40 female LDLR(−/−) mice (8–12 weeks-old) were fed a western-type diet (0.25% cholesterol, 15% fatty acids; Arie Blok, Woerden, the Netherlands) for 12 weeks to induce NASH and 10 mice were sacrificed. The 30 remaining mice were subdivided into 3 groups of 10 animals, one group continued the same diet for another 6 weeks, the second group received the same diet supplemented with 0.3 g AMP-DNM per kg diet to obtain the calculated dose of 50 mg AMP-DNM. kg bw^−1^. day^−1^ and the third group received 0.6 g AMP-DNM per kg diet to obtain 100 mg AMP-DNM. kg bw^−1^. day^−1^. In parallel, one group of 5 LDLR(−/−) mice was kept on chow diet. In a second experiment, 40 female APOE*3 Leiden (8–12 weeks-old) were fed a high fat-high cholesterol diet (1% cholesterol, 15% fatty acids) for 12 weeks and 10 mice were sacrificed. The 30 remaining mice continued with a western-type diet (0.25% cholesterol, 15% fatty acids) supplemented or not with two different doses of AMP-DNM to obtain the calculated dose of 50 mg and 100 mg AMP-DNM. kg bw^−1^. day^−1^.

### Plasma and tissue sampling

Blood samples of non-fasted animals were collected via the tail vein at the time points indicated in the figures. At the end of the experiments, blood samples were collected by abdominal aorta puncture and plasma samples were stored at −20°C. Livers were quickly excised and weighed, and parts were snap-frozen in liquid nitrogen and stored at −80°C, or fixed in 10% buffered formalin and embedded in paraffin.

### Analytical procedures

Blood glucose levels were determined in plasma of fasted animals using a hand-held Glucometer (Ascensia Elite; Bayer A.G., Leverkusen, Germany) one week before the sacrifice of the animals. Hemoglobin A1c levels were measured using a single measurement A1C now device (Metrika, Sunnyvale, CA). Insulin levels were determined by enzyme-linked immunosorbent assay (Crystal Chem Inc., Downers Grove, IL). Free cholesterol, total cholesterol, and triglycerides in liver samples were determined after lipid extraction according to Folch [41] using a colorimetric enzymatic kit (Biolabo, Maizy, France). Cholesterol and triglyceride levels in plasma were determined using the same assay. FFA level in plasma were determined using a colorimetric enzymatic kit (Wako Chemicals Gmbh, Neuss, Germany). Ceramide and GlcCer were determined after Folch extraction as previously describe [11]. Plasma cholesterol concentrations in the main lipoprotein classes were determined in pooled plasma samples of each group (5 mice) separated by high performance gel filtration chromatography [42].

### Histology

Paraffin embedded liver sections (7 µM) were de-waxed and stained with hematoxylin-eosin for general histology, or with 0.2% picro-sirius red to detect fibrillar collagen deposits. To detect neutral lipids, cryostat sections of 7 µM were stained with 0.3% Oil Red O. For detection of macrophages/monocytes, a rat polyclonal anti-CD68 was used (Serotec, Oxford UK) and an anti-alpha smooth muscle actin (SMA) antibody to visualize activated stellate cells (1A4, Abcam, Cambridge, UK). Frozen sections of livers fixed in cold acetone were incubated one hour at room temperature with CD68 (1/100 dilution) or SMA antibody (1/100 dilution) followed by incubation for 30 min at room temperature with the corresponding secondary antibodies. Visualization of the complex was done with 3,3′-diaminobenzidine tetrahydrochloride (DAB, Immunologic, Duiven, The Netherlands) for 5 min. For all stainings, haematoxylin (Sigma-Aldrich, Zwijndrecht, the Netherlands) was used to counterstain. Primary antibodies were omitted in negative control samples. Images were captured with a Leica DFC 420 video camera.

### Determination of mRNA levels

Total RNA was isolated from 50–100 mg of liver tissue using Trizol reagent (Invitrogen, Breda, The Netherlands) and reverse transcribed with SuperScript II Reverse Transcriptase and random hexamers (Invitrogen, Breda, The Netherlands) after treatment with RQ1 Rnase-free Dnase (1 units/2 µg of total RNA, Promega, Leiden, The Netherlands). Gene expression analysis was performed on a Bio-Rad MyIQ Single-color Real-Time PCR Detection System by using the Bio-Rad IQ SYBR Green Supermix (Bio-Rad Laboratories Inc., Hercules, CA). Expression levels were normalized to Acidic ribosomal phosphoprotein (36B4).

### Statistical analysis

Values presented in figures concerning the LDLR(−/−) mice represent mean ± SEM. Dunnett's comparison test was performed between 18 weeks controls and each other group using GraphPad Prism software. Values presented in figures concerning the APOE*3 Leiden mice represent mean ± SEM. Dunnett's comparison test was performed between baseline at 12 weeks and each other group using GraphPad Prism software *, p<0.05; **, p<0.01; ***, P<0.001.

## Supporting Information

Figure S1 Genes expression in livers of APOE*3 Leiden mice fed a high cholesterol-high fat diet (1% cholesterol, 15% fat) for 12 weeks and fed for 6 more weeks a western-type diet (0.25% cholesterol, 15% fat) supplemented with either 0, 50 or 100 mg AMP-DNM. Expression levels normalized to Acidic ribosomal phosphoprotein (36B4). Data are expressed as mean ± SEM, n = 5. Statistical significance determined between baseline 12w and other groups with Dunnett's comparison test. *p<0.05, **p<0.01, p<0.001.
(DOC)

Figure S2 Effect of AMP-DNM treatment on insulin sensitivity in APOE*3 Leiden mice fed a high cholesterol-high fat diet (1% cholesterol, 15% fat) for 12 weeks and fed for 6 more weeks a western-type diet (0.25% cholesterol, 15% fat) supplemented with either 0, 50 or 100 mg AMP-DNM. Data are expressed as mean ± SEM, n = 5. Statistical significance determined between baseline 12w and other groups with Dunnett's comparison test. *p<0.05, **p<0.01, p<0.001.
(DOC)

Figure S3 **FACs analysis of leucocytes isolated from livers of LDLR(−/−) mice fed a western-type diet for 18 weeks, receiving in the last 6 weeks either 0, 50 or 100 mg AMP-DNM.** (A) CD3 positive cells and natural killer T (NKT) cells (NK1.1 and CD3 double positive cells). (B) CD4 and CD8 positive cells. (C) F4/80 positive cells. Data are expressed as mean ± SEM, n = 4. Statistical significance between control and treated groups was determined by Dunnett's comparison test **p<0.01; *p<0.05.
(DOC)

Table S1 **Effect of AMP-DNM treatment on bodyweight and food intake in LDLR(−/−) mice fed a western-type diet for 18 weeks, receiving in the last 6 weeks either 0, 50 or 100 mg AMP-DNM.** Data are expressed as mean ± SEM, n = 10 for bodyweight. Food intake based on the amount of food left in each cage of treatment (2 to 3 cages per treatment) at the end of each dosing week.
(DOC)

Table S2 Effect of AMP-DNM treatment on bodyweight and food intake in APOE*3 Leiden mice fed a high cholesterol-high fat diet (1% cholesterol, 15% fat) for 12 weeks and fed for 6 following weeks a western-type diet (0.25% cholesterol, 15% fat) supplemented with either 0, 50 or 100 mg AMP-DNM. Data are expressed as mean ± SEM, n = 5 for bodyweight. Food intake based on the amount of food left in each cage of treatment (2 to 3 cages per treatment) at the end of each dosing week.
(DOC)

Table S3 Plasma lipids concentrations in APOE*3 Leiden mice fed a high cholesterol-high fat diet (1% cholesterol, 15% fat) for 12 weeks and fed for 6 more weeks a western-type diet (0.25% cholesterol, 15% fat) supplemented with either 0, 50 or 100 mg AMP-DNM. Data are expressed as mean ± SEM, n = 5.
(DOC)

Table S4 Liver glycosphingolipids concentrations in APOE*3 Leiden mice fed a high cholesterol-high fat diet (1% cholesterol, 15% fat) for 12 weeks and fed for 6 more weeks a western-type diet (0.25% cholesterol, 15% fat) supplemented with either 0, 50 or 100 mg AMP-DNM. Data are expressed as mean ± SEM, n = 5.
(DOC)

## References

[1] Varela-Rey M, Embade N, Ariz U, Lu SC, Mato JM, et al (2009) Non-alcoholic steatohepatitis and animal models: understanding the human disease. Int J Biochem Cell Biol 41: 969–976. 19027869 10.1016/j.biocel.2008.10.027

[2] McCullough AJ (2006) Pathophysiology of nonalcoholic steatohepatitis. J Clin Gastroenterol 40 Suppl 1: S17–S29. 16540762 10.1097/01.mcg.0000168645.86658.22

[3] Solga SF, Alkhuraishe A, Cope K, Tabesh A, Clark JM, et al (2006) Breath biomarkers and non-alcoholic fatty liver disease: preliminary observations. Biomarkers 11: 174–183. 16766393 10.1080/13547500500421070

[4] Ekstedt M, Franzen LE, Mathiesen UL, Thorelius L, Holmqvist M, et al (2006) Long-term follow-up of patients with NAFLD and elevated liver enzymes. Hepatology 44: 865–873. 17006923 10.1002/hep.21327

[5] Lewis JR, Mohanty SR (2010) Nonalcoholic fatty liver disease: a review and update. Dig Dis Sci 55: 560–578. 20101463 10.1007/s10620-009-1081-0

[6] Wouters K, van Gorp PJ, Bieghs V, Gijbels MJ, Duimel H, et al (2008) Dietary cholesterol, rather than liver steatosis, leads to hepatic inflammation in hyperlipidemic mouse models of nonalcoholic steatohepatitis. Hepatology 48: 474–486. 18666236 10.1002/hep.22363

[7] Vinaixa M, Rodriguez MA, Rull A, Beltran R, Blade C, et al (2010) Metabolomic assessment of the effect of dietary cholesterol in the progressive development of fatty liver disease. J Proteome Res 9: 2527–2538. 20402505 10.1021/pr901203w

[8] Zhao H, Przybylska M, Wu IH, Zhang J, Siegel C, et al (2007) Inhibiting glycosphingolipid synthesis improves glycemic control and insulin sensitivity in animal models of type 2 diabetes. Diabetes 56: 1210–1218. 17470562 10.2337/db06-0719

[9] Bijl N, Sokolovic M, Vrins C, Langeveld M, Moerland PD, et al (2009) Modulation of glycosphingolipid metabolism significantly improves hepatic insulin sensitivity and reverses hepatic steatosis in mice. Hepatology 50: 1431–1441. 19731235 10.1002/hep.23175

[10] Yew NS, Zhao H, Hong EG, Wu IH, Przybylska M, et al (2010) Increased hepatic insulin action in diet-induced obese mice following inhibition of glucosylceramide synthase. PLoS One 5: e11239. 20574539 10.1371/journal.pone.0011239 PMC2888613

[11] Aerts JM, Ottenhoff R, Powlson AS, Grefhorst A, van Eijk M, et al (2007) Pharmacological inhibition of glucosylceramide synthase enhances insulin sensitivity. Diabetes 56: 1341–1349. 17287460 10.2337/db06-1619 PMC4298701

[12] van Eijk M, Aten J, Bijl N, Ottenhoff R, van Roomen CP, et al (2009) Reducing glycosphingolipid content in adipose tissue of obese mice restores insulin sensitivity, adipogenesis and reduces inflammation. PLoS One 4: e4723. 19305508 10.1371/journal.pone.0004723 PMC2654925

[13] Zhao H, Przybylska M, Wu IH, Zhang J, Maniatis P, et al (2009) Inhibiting glycosphingolipid synthesis ameliorates hepatic steatosis in obese mice. Hepatology 50: 85–93. 19444873 10.1002/hep.22970

[14] Yamashita T, Hashiramoto A, Haluzik M, Mizukami H, Beck S, et al (2003) Enhanced insulinsensitivity in mice lacking ganglioside GM3. Proc Natl Acad Sci U S A 100: 3445–3449. 12629211 10.1073/pnas.0635898100 PMC152312

[15] Kabayama K, Sato T, Saito K, Loberto N, Prinetti A, et al (2007) Dissociation of the insulin receptor and caveolin-1 complex by ganglioside GM3 in the state of insulin resistance. Proc Natl Acad Sci U S A 104: 13678–13683. 17699617 10.1073/pnas.0703650104 PMC1949342

[16] Wennekes T, van den Berg RJ, Boot RG, van der Marel GA, Overkleeft HS, et al (2009) Glycosphingolipids–nature, function, and pharmacological modulation. Angew Chem Int Ed Engl 48: 8848–8869. 19862781 10.1002/anie.200902620

[17] Langeveld M, Aerts JM (2009) Glycosphingolipids and insulin resistance. Prog Lipid Res 48: 196–205. 19303901 10.1016/j.plipres.2009.03.002

[18] Wennekes T, Meijer AJ, Groen AK, Boot RG, Groener JE, et al (2010) Dual-action lipophilic iminosugar improves glycemic control in obese rodents by reduction of visceral glycosphingolipids and buffering of carbohydrate assimilation. J Med Chem 53: 689–698. 20000679 10.1021/jm901281m

[19] Brown MS, Goldstein JL (2008) Selective versus total insulin resistance: a pathogenic paradox. Cell Metab 7: 95–96. 18249166 10.1016/j.cmet.2007.12.009

[20] Ripoll VM, Irvine KM, Ravasi T, Sweet MJ, Hume DA (2007) Gpnmb is induced in macrophages by IFN-gamma and lipopolysaccharide and acts as a feedback regulator of proinflammatory responses. J Immunol 178: 6557–6566. 17475886 10.4049/jimmunol.178.10.6557

[21] Larter CZ, Yeh MM (2008) Animal models of NASH: getting both pathology and metabolic context right. J Gastroenterol Hepatol 23: 1635–1648. 18752564 10.1111/j.1440-1746.2008.05543.x

[22] Rull A, Rodriguez F, Aragones G, Marsillach J, Beltran R, et al (2009) Hepatic monocyte chemoattractant protein-1 is upregulated by dietary cholesterol and contributes to liver steatosis. Cytokine 48: 273–279. 19748796 10.1016/j.cyto.2009.08.006

[23] Bietrix F, Lombardo E, van Roomen CP, Ottenhoff R, Vos M, et al (2010) Inhibition of glycosphingolipid synthesis induces a profound reduction of plasma cholesterol and inhibits atherosclerosis development in APOE*3 Leiden and low-density lipoprotein receptor−/− mice. Arterioscler Thromb Vasc Biol 30: 931–937. 20167657 10.1161/ATVBAHA.109.201673

[24] Gastaldelli A, Harrison S, Belfort-Aguiar R, Hardies J, Balas B, et al (2010) Pioglitazone in the treatment of NASH: the role of adiponectin. Aliment Pharmacol Ther 32: 769–775. 20662773 10.1111/j.1365-2036.2010.04405.x

[25] Langeveld M, van den Berg SA, Bijl N, Bijland S, van Roomen CP, et al (2012) Treatment of genetically obese mice with the iminosugar N-(5-adamantane-1-yl-methoxy-pentyl)-deoxynojirimycin reduces body weight by decreasing food intake and increasing fat oxidation. Metabolism 61: 99–107. 21816446 10.1016/j.metabol.2011.05.013

[26] Tsuduki T, Nakamura Y, Honma T, Nakagawa K, Kimura T, et al (2009) Intake of 1-deoxynojirimycin suppresses lipid accumulation through activation of the beta-oxidation system in rat liver. J Agric Food Chem 57: 11024–11029. 19863049 10.1021/jf903132r

[27] Kobayashi Y, Miyazawa M, Kamei A, Abe K, Kojima T (2010) Ameliorative effects of mulberry (Morus alba L.) leaves on hyperlipidemia in rats fed a high-fat diet: induction of fatty acid oxidation, inhibition of lipogenesis, and suppression of oxidative stress. Biosci Biotechnol Biochem 74: 2385–2395. 21150120 10.1271/bbb.100392

[28] Jennemann R, Rothermel U, Wang S, Sandhoff R, Kaden S, et al (2010) Hepatic glycosphingolipid deficiency and liver function in mice. Hepatology 51: 1799–1809. 20432257 10.1002/hep.23545

[29] Shen C, Bullens D, Kasran A, Maerten P, Boon L, et al (2004) Inhibition of glycolipid biosynthesis by N-(5-adamantane-1-yl-methoxy-pentyl)-deoxynojirimycin protects against the inflammatory response in hapten-induced colitis. Int Immunopharmacol 4: 939–951. 15182733 10.1016/j.intimp.2004.04.008

[30] Lee AH, Scapa EF, Cohen DE, Glimcher LH (2008) Regulation of hepatic lipogenesis by the transcription factor XBP1. Science 320: 1492–1496. 18556558 10.1126/science.1158042 PMC3620093

[31] Ozcan U, Cao Q, Yilmaz E, Lee AH, Iwakoshi NN, et al (2004) Endoplasmic reticulum stress links obesity, insulin action, and type 2 diabetes. Science 306: 457–461. 15486293 10.1126/science.1103160

[32] Ozcan L, Tabas I (2012) Role of endoplasmic reticulum stress in metabolic disease and other disorders. Annu Rev Med 63: 317–328. 22248326 10.1146/annurev-med-043010-144749 PMC3290993

[33] Boslem E, MacIntosh G, Preston AM, Bartley C, Busch AK, et al (2011) A lipidomic screen of palmitate-treated MIN6 β-cells links sphingolipid metabolites with endoplasmic reticulum (ER) stress and impaired protein trafficking. Biochem J 435: 267–76. 21265737 10.1042/BJ20101867

[34] Li Z, Soloski MJ, Diehl AM (2005) Dietary factors alter hepatic innate immune system in mice with nonalcoholic fatty liver disease. Hepatology 42: 880–885. 16175608 10.1002/hep.20826

[35] Kremer M, Thomas E, Milton RJ, Perry AW, van RN, et al (2010) Kupffer cell and interleukin-12-dependent loss of natural killer T cells in hepatosteatosis. Hepatology 51: 130–141. 20034047 10.1002/hep.23292 PMC3761962

[36] Marí M, Caballero F, Colell A, Morales A, Caballeria J, et al (2006) Mitochondrial free cholesterol loading sensitizes to TNF- and Fas-mediated steatohepatitis. Cell Metab 4: 185–198. 16950136 10.1016/j.cmet.2006.07.006

[37] Van Rooyen DM, Larter CZ, Haigh WG, Yeh MM, Ioannou G, et al (2011) Hepatic free cholesterol accumulates in obese, diabetic mice and causes nonalcoholic steatohepatitis. Gastroenterology 141: 1393–1403. 21703998 10.1053/j.gastro.2011.06.040 PMC3186822

[38] Bieghs V, Wouters K, van Gorp PJ, Gijbels MJ, de Winther MP, et al (2010) Role of scavenger receptor A and CD36 in diet-induced nonalcoholic steatohepatitis in hyperlipidemic mice. Gastroenterology 138: 2477–2486, 2477-86-2486. 20206177 10.1053/j.gastro.2010.02.051 PMC3114629

[39] Overkleeft HS, Renkema GH, Neele J, Vianello P, Hung IO, et al (1998) Generation of specific deoxynojirimycin-type inhibitors of the non-lysosomal glucosylceramidase. J Biol Chem 273: 26522–26527. 9756888 10.1074/jbc.273.41.26522

[40] Wennekes T, van den Berg RJ, Donker W, van der Marel GA, Strijland A, et al (2007) Development of adamantan-1-yl-methoxy-functionalized 1-deoxynojirimycin derivatives as selective inhibitors of glucosylceramide metabolism in man. J Org Chem 72: 1088–1097. 17243712 10.1021/jo061280p

[41] Folch J, Lees M, Sloane Stanley GH (1957) A simple method for the isolation and purification of total lipides from animal tissues. J Biol Chem 226: 497–509. 13428781

[42] Levels JH, Lemaire LC, van den Ende AE, van Deventer SJ, van Lanschot JJ (2003) Lipid composition and lipopolysaccharide binding capacity of lipoproteins in plasma and lymph of patients with systemic inflammatory response syndrome and multiple organ failure. Crit Care Med 31: 1647–1653. 12794399 10.1097/01.CCM.0000063260.07222.76

