# Supplementary figures and images for: Correction of Liver Steatosis by a Hydrophobic Iminosugar Modulating Glycosphingolipids Metabolism
Source: PLoS One. 2012 Oct 8;7(10):e38520. doi: 10.1371/journal.pone.0038520 (PMC3466229; doi:10.1371/journal.pone.0038520)

**Figure S1**


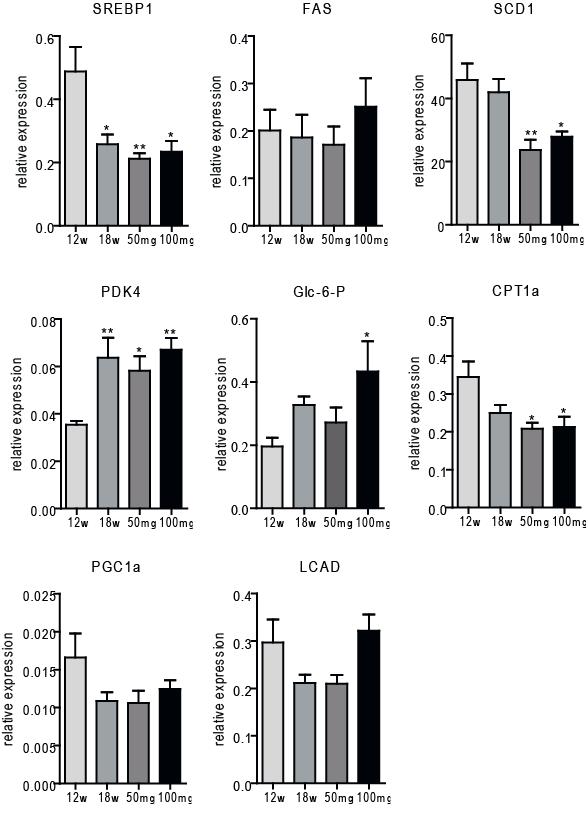

Supplement: Figure S1 — Genes expression in livers of APOE*3 Leiden mice fed a high cholesterol-high fat diet (1% cholesterol, 15% fat) for 12 weeks and fed for 6 more weeks a western-type diet (0.25% cholesterol, 15% fat) supplemented with either 0, 50 or 100 mg AMP-DNM. Expression levels normalized to Acidic ribosomal phosphoprotein (36B4). Data are expressed as mean ± SEM, n = 5. Statistical significance determined between baseline 12w and other groups with Dunnett's comparison test. *p<0.05, **p<0.01, p<0.001. (DOC) [file pone.0038520.s001.doc]

**Figure S2:**


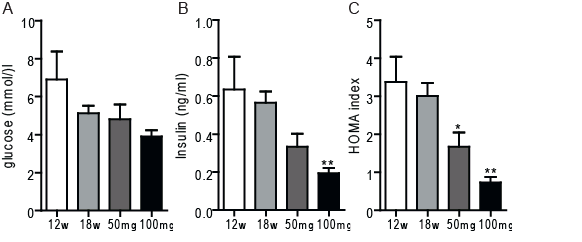

Supplement: Figure S2 — Effect of AMP-DNM treatment on insulin sensitivity in APOE*3 Leiden mice fed a high cholesterol-high fat diet (1% cholesterol, 15% fat) for 12 weeks and fed for 6 more weeks a western-type diet (0.25% cholesterol, 15% fat) supplemented with either 0, 50 or 100 mg AMP-DNM. Data are expressed as mean ± SEM, n = 5. Statistical significance determined between baseline 12w and other groups with Dunnett's comparison test. *p<0.05, **p<0.01, p<0.001. (DOC) [file pone.0038520.s002.doc]

**Figure S3**


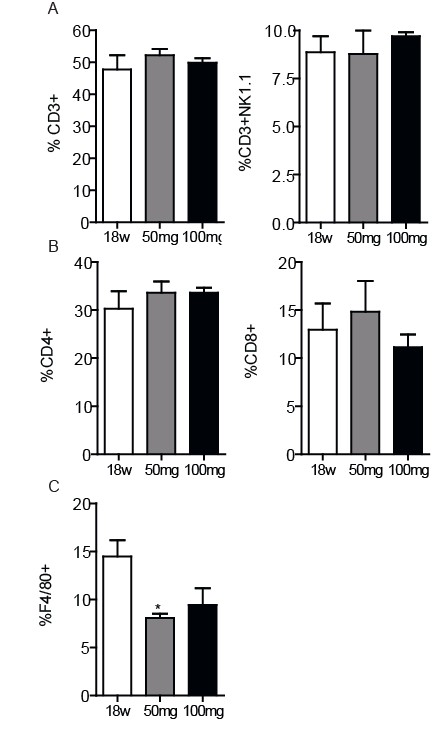

Supplement: Figure S3 — FACs analysis of leucocytes isolated from livers of LDLR(−/−) mice fed a western-type diet for 18 weeks, receiving in the last 6 weeks either 0, 50 or 100 mg AMP-DNM. (A) CD3 positive cells and natural killer T (NKT) cells (NK1.1 and CD3 double positive cells). (B) CD4 and CD8 positive cells. (C) F4/80 positive cells. Data are expressed as mean ± SEM, n = 4. Statistical significance between control and treated groups was determined by Dunnett's comparison test **p<0.01; *p<0.05. (DOC) [file pone.0038520.s003.doc]
