# Supplementary material for: Correction of Liver Steatosis by a Hydrophobic Iminosugar Modulating Glycosphingolipids Metabolism
Source: PLoS One. 2012 Oct 8;7(10):e38520. doi: 10.1371/journal.pone.0038520 (PMC3466229; doi:10.1371/journal.pone.0038520)
Supplement: Table S1 — Effect of AMP-DNM treatment on bodyweight and food intake in LDLR(−/−) mice fed a western-type diet for 18 weeks, receiving in the last 6 weeks either 0, 50 or 100 mg AMP-DNM. Data are expressed as mean ± SEM, n = 10 for bodyweight. Food intake based on the amount of food left in each cage of treatment (2 to 3 cages per treatment) at the end of each dosing week. (DOC) [file pone.0038520.s004.doc]

**Table S1**

| **Bodyweight (g)** | | | | |
| --- | --- | --- | --- | --- |
|  | **12w (baseline)** | **CTRL** | **50mg** | **100mg** |
| **12w** | 22.3±0.5 | 21.8±0.4 | 21.5±0.2 | 21.4±0.4 |
| **18w** | xxx | 21.9±0.2 | 21.9±0.1 | 20.1±0.2*** |
| **Food intake (g/24h/100g bodyweight)** | | | | |
| **12w** | 11.3 | 11.5±1.5 | 11.9±0.9 | 9.7 |
| **18w** | xxx | 12.7±2.6 | 15.4±3.2 | 9.9 |

***p<0.001, statistical significance determined between baseline 12w and others groups with Dunnett’s comparison test.
