# Supplementary material for: Correction of Liver Steatosis by a Hydrophobic Iminosugar Modulating Glycosphingolipids Metabolism
Source: PLoS One. 2012 Oct 8;7(10):e38520. doi: 10.1371/journal.pone.0038520 (PMC3466229; doi:10.1371/journal.pone.0038520)
Supplement: Table S2 — Effect of AMP-DNM treatment on bodyweight and food intake in APOE*3 Leiden mice fed a high cholesterol-high fat diet (1% cholesterol, 15% fat) for 12 weeks and fed for 6 following weeks a western-type diet (0.25% cholesterol, 15% fat) supplemented with either 0, 50 or 100 mg AMP-DNM. Data are expressed as mean ± SEM, n = 5 for bodyweight. Food intake based on the amount of food left in each cage of treatment (2 to 3 cages per treatment) at the end of each dosing week. (DOC) [file pone.0038520.s005.doc]

**Table S2**

| **Bodyweight (g)** | | | | |
| --- | --- | --- | --- | --- |
|  | **12w (baseline)** | **CTRL** | **50mg** | **100mg** |
| **12w** | 23.6±0.9 | 23.5±0.5 | 23.8±0.5 | 25.1±0.6 |
| **18w** | xxx | 25.5±0.7 | 23.0±0.4* | 22.9±0.5* |
| **Food intake (g/24h/100g bodyweight)** | | | | |
| **12w** | 11.1±0.5 | 10.1±0.9 | 10.2±0.4 | 9.4 |
| **18w** | xxx | 9.3±0.4 | 11.7±0.6 | 11.5 |

*p<0.05, statistical significance determined between baseline 12w and others groups and treated groups with Dunnett’s comparison test.
