# Supplementary material for: Correction of Liver Steatosis by a Hydrophobic Iminosugar Modulating Glycosphingolipids Metabolism
Source: PLoS One. 2012 Oct 8;7(10):e38520. doi: 10.1371/journal.pone.0038520 (PMC3466229; doi:10.1371/journal.pone.0038520)
Supplement: Table S3 — Plasma lipids concentrations in APOE*3 Leiden mice fed a high cholesterol-high fat diet (1% cholesterol, 15% fat) for 12 weeks and fed for 6 more weeks a western-type diet (0.25% cholesterol, 15% fat) supplemented with either 0, 50 or 100 mg AMP-DNM. Data are expressed as mean ± SEM, n = 5. (DOC) [file pone.0038520.s006.doc]

**Table S3**

|  | **12w** | **CTRL** | **50mg** | **100mg** |
| --- | --- | --- | --- | --- |
| **glccer**  (nmol/ml) | 43.9±6.9 | 51.7±8.8 | 12.8±1.9** | 3.6±0.2*** |
| **cer**  (nmol/ml) | 19.9±3.3 | 25.2±4.0 | 11.3±0.8 | 3.0±0.2*** |
| **cholesterol**  (mmol/ml) | 22.2±1.6 | 17.8±1.4 | 8.9±0.7*** | 3.3±0.3*** |
| **triglycerides**  (mmol/ml) | 3.8±0.2 | 3.3±0.2 | 1.6±0.3* | 0.7±0.1*** |

*p<0.05; **p<0.01; ***p<0.001, statistical significance between baseline 12w and others groups and treated groups with Dunnett’s comparison test.
