# Supplementary material for: Correction of Liver Steatosis by a Hydrophobic Iminosugar Modulating Glycosphingolipids Metabolism
Source: PLoS One. 2012 Oct 8;7(10):e38520. doi: 10.1371/journal.pone.0038520 (PMC3466229; doi:10.1371/journal.pone.0038520)
Supplement: Table S4 — Liver glycosphingolipids concentrations in APOE*3 Leiden mice fed a high cholesterol-high fat diet (1% cholesterol, 15% fat) for 12 weeks and fed for 6 more weeks a western-type diet (0.25% cholesterol, 15% fat) supplemented with either 0, 50 or 100 mg AMP-DNM. Data are expressed as mean ± SEM, n = 5. (DOC) [file pone.0038520.s007.doc]

**Table S4**

|  | **12w** | **CTRL** | **50mg** | **100mg** |
| --- | --- | --- | --- | --- |
| **glccer**  (nmol/g liver) | 133.4±5.5 | 136.0±5.5 | 73.6±6.0*** | 72.9±5.8*** |
| **cer**  (nmol/g liver) | 429.5±18.3 | 470.3±25.5 | 472.7±38.9* | 489.6±14.4*** |

*p<0.05; ***p<0.001, statistical significance between baseline 12w and others groups and treated groups with Dunnett’s comparison test.
